# Intestinal Invagination

**Published:** 1874-11

**Authors:** 


DIFFERENTIATION OF INTESTINAL INVAGINATION.—Dr. O. Leichtenstein, in an article on invagination (*Archiv f. Prakt. Heilk.*, 4, 1873,) refers to the following points for the differentiation of invagination of the small from that of the large intestine: I. Invagination of the small intestine but rarely occurs during the first year of life, as also rarely during childhood in general. 2. In adults, the course of the attack in invagination of the ileum is more rapid, the phenomena more severe, than in ileo-cæcal and colon invaginations. Chronic cases are rare in invaginations of the small intestine, more frequent in those of the ileo-cæcum and colon. Severe symptoms of collapse occur more frequently in the beginning of the disease. 3. Muco-sanguinolent discharges are the rule in all invaginations, whatever their seat. Fæcal evacuations, entirely normal in character, (after preceding diarrhœa) were observed in ileo-cæcal invaginations, once in a colon invagination, the patient being an adult. 4. Meteorism is a very variable symptom. It is usually absent in ileo-cæcal invaginations. In invaginations of the descending colon it was frequently recognized as affecting the transverse colon, and subsequently spread over the whole abdomen. In invagination of the ileum it was occasionally found to be confined principally to the central abdominal region, with exemption of the lateral portions and epigastrium. 5. Tenesmus is rare in invagination of the ileum, frequent in that of the colon and ileo-cæcum. 6. The tumor is usually absent in ileum invaginations. Its seat in the center of the hypogastrium speaks for the variety; when situated in the cæcal region, especially when it remains stationary for some time, it indicates ileum or ileo-cæcal invagination. The spread of the tumor, when occurring suddenly and corresponding to the course of the colon, speaks more for ileo-cæcal, less for colon invagination, and excludes ileum invagination. The seat of the tumor in the left lateral portions of the abdomen would indicate ileo-cæcal or colon invagination. The tumor can never be felt in the rectum, and prolapse through the latter never occurs in uncomplicated ileum invagination. Changes in the consistency, occurrence, and disappearance of the tumor were especially observed in ileo-cæcal invagination.—*New York Med. Journal, Sept.,* 1874.

